# Neuronal and Glial α7 Nicotinic Acetylcholine Receptors: Role in Alzheimer’s Disease Pathophysiology

**DOI:** 10.3390/life15071032

**Published:** 2025-06-28

**Authors:** Kerry Rennie

**Affiliations:** Human Health Therapeutics, National Research Council, Ottawa, ON K1A 0R6, Canada; kerry.rennie@nrc-cnrc.gc.ca

**Keywords:** Alzheimer’s disease, alpha7 nicotinic acetylcholine receptor, beta amyloid

## Abstract

Cholinergic projections from the basal forebrain to the cortex and hippocampus play a critical role in cognitive functions, many of which rely on signaling through the alpha7 nicotinic acetylcholine receptor (α7nAChR). The Alzheimer’s disease (AD) brain is characterized by the profound impairment of the basal forebrain cholinergic system, including alterations in the levels of α7nAChR in various brain areas. In addition, α7nAChR binds with high affinity to beta amyloid (Aβ), suggesting α7nAChR might mediate some of Aβ’s effects in the brain. Under normal physiological conditions, the interaction between Aβ and α7nAChR appears to be beneficial, supporting normal neurotransmission, synaptic plasticity, and memory functions. However, when levels of Aβ are pathologically elevated, their interaction leads to deleterious effects, implicating α7nAChR in the pathophysiology of AD. In addition to expression in neurons, α7nAChR is expressed in astrocytes and microglia, where it serves as a key component of a cholinergic pathway that regulates neuroinflammation. This review article will cover the role of α7nAChR in neurons, astrocytes and microglia under normal conditions, summarize changes in the expression or function of α7nAChR in neurons and glia in the AD brain, and discuss cell-type specific contributions of α7nAChR to AD pathology with an emphasis on interactions of α7nAChR with Aβ.

## 1. Introduction

Alzheimer’s disease (AD) is distinguished neuropathologically by the presence of extracellular plaques containing misfolded beta-amyloid (Aβ), as well as aberrant intracellular inclusions of hyperphosphorylated tau protein in the form of neurofibrillary tangles [1]. Other notable features include loss of synapses, preceding outright neuronal loss, and neuroinflammation [1]. In addition, extensive loss of cholinergic innervation to the hippocampus and cortex is a characteristic feature of the AD brain [2].

The basal forebrain cholinergic system (BFCS), composed of acetylcholine (ACh)-producing neurons in the medial septum, vertical and horizontal bands of the diagonal band of Broca, and the nucleus basalis of Meynert, is the major source of cholinergic innervation to the cortex, hippocampus, amygdala, and olfactory bulb [2,3]. ACh acts as a neuromodulator in the brain, influencing activity in target areas by modifying neuronal excitability, adjusting neurotransmitter release, and coordinating activity across groups of neurons [4,5]. ACh also modulates synaptic plasticity, resulting in long-lasting changes in neuronal communication [5,6]. Through these actions, basal forebrain cholinergic projections to the hippocampus and cortex play a vital role in several cognitive functions including acquisition, encoding, consolidation, extinction, and retrieval of memory as well as top-down attentional processing [7,8,9,10].

Early studies indicated that postmortem AD brains exhibit reduced activity of choline acetyltransferase, the enzyme responsible for ACh synthesis, in brain regions targeted by the BFCS [11] as well as loss of magnocellular neurons in the basal forebrain [12]. These discoveries, along with findings that pharmacological manipulation of the cholinergic system resulted in cognitive impairments [13], led to the development of the cholinergic hypothesis of Alzheimer’s disease (reviewed in [14,15]) which posits that dysfunction of basal forebrain cholinergic neurons underlies cognitive decline in AD. The cholinergic hypothesis predicted that boosting cholinergic function should improve cognition and led to the clinical development of cholinesterase inhibitors, which prevent the enzymatic breakdown of ACh, as the first approved treatments for AD [16]. Notably, recent brain imaging studies suggest that damage to the basal forebrain occurs early in the course of AD and both precedes and predicts degeneration of the entorhinal cortex (which in turn predicts degeneration of other cortical areas) as well as cognitive decline [17,18,19]. These findings further emphasize the importance of the cholinergic system in the pathogenesis of AD and the potential for targeting the cholinergic system as disease-modifying therapy.

## 2. Cholinergic Receptors

ACh released from basal forebrain axonal projections acts on two families of receptors–muscarinic (mAChR) and nicotinic (nAChR). Muscarinic receptors are G-protein coupled 7 transmembrane domain metabotropic receptors and have excitatory or inhibitory neuromodulatory effects depending on the subtype. M1, M3, and M5 receptors mediate excitatory actions via interaction with G_q/11_ G-proteins, whereas M2 and M4 subtypes have inhibitory effects by signaling through G_i/o_ G-proteins [20]. Nicotinic receptors are ionotropic and are formed as an assembly of five subunits creating a central cation permeable channel [21]. The pentamers are made up of combinations of various alpha (α2–α10) and/or beta (β2–β4) subunits and can be either homomeric, containing a single subunit type (e.g., α7 or α9), or heteromeric, containing combinations of different α-subunits, or α- and β-subunits [22]. Each subunit has a large extracellular N terminal domain housing the ACh binding site, four transmembrane domains (M1-M4), a large intracellular loop between M3 and M4, and an extracellular C-terminal tail [23]. The ion channel is lined by the M2 domain, which contains residues that influence ion permeability, selectivity, and channel gating [24]. The particular composition of subunits within the receptor confers different functional properties, including affinity for ACh and other agonists and antagonists, channel kinetics, and permeability to different cations [24].

While various muscarinic and nicotinic receptor subtypes have been implicated in AD, here, the focus will be on α7-subunit-containing receptors, including the homomeric α7 receptor and the more recently described heteromeric α7β2 receptor. The α7 receptor is of interest owing to its prominent role in mediating cognitive processes as well as evidence linking α7 to Aβ-induced pathology.

## 3. The α7 Nicotinic Receptor

A number of properties distinguish the homomeric α7nAChR from other nAChR subtypes, including relatively low affinity for ACh and nicotine, short open time, rapid and long-lasting desensitization, and high permeability to Ca^2+^ [25,26]. Ca^2+^ serves as a versatile second messenger that links α7nAChR activation to intracellular signaling pathways, affecting the expression and function of many genes and proteins [26]. However, since excessive Ca^2+^ influx can be damaging to cells, the short open time and rapid desensitization of α7nAChR serve to limit Ca^2+^ influx and subsequent excitotoxicity [26].

Increased intracellular Ca^2+^ levels upon α7nAChR activation can result from the direct influx of Ca^2+^ through the α7nAChR channel, as well as from influx through voltage-gated Ca^2+^ channels (VGCCs) which become activated in response to depolarization driven by nAChR activation [22]. Furthermore, Ca^2+^ released from intracellular stores via ryanodine receptors (stimulated by Ca^2+^ influx) can contribute to elevated Ca^2+^ levels following α7nAChR activation [22,27]. α7nAChR also have metabotropic-like functions through their interactions with various G-proteins and can stimulate inositol phosphate-induced Ca^2+^ release from intracellular stores [27,28]. The extent to which these mechanisms are employed is dependent on cell type, with α7nAChR in some cell types seeming to operate exclusively in metabotropic mode while others use a combination of metabotropic and ionotropic signaling [27]. Interestingly, metabotropic responses of α7nAChR can be induced by both agonists and antagonists [27,29], and an additional class of α7nAChR ligands has been identified, termed silent agonists, which stabilize the receptor in a desensitized state enabling metabotropic but not ionotropic signaling [30]. Amplification of intracellular Ca^2+^ by release from intracellular stores and activation of G-protein-dependent signaling cascades produces changes that persist beyond channel activation and into the desensitized state [27], increasing the diversity and longevity of responses that can be initiated by α7nAChR binding.

Several neuronal subtypes express α7nAChR, including both excitatory and inhibitory neurons [31,32]. α7nAChR may be found pre-, post- and perisynaptically and can localize to dendrites, axons, and soma, with the subcellular distribution being dependent on neuronal subtype [32,33,34]. Presynaptic α7nAChR can facilitate Ca^2+^-dependent release of neurotransmitters in different areas of the brain, including glutamate, gamma-aminobutyric acid (GABA), and dopamine [35,36] and can therefore have excitatory or inhibitory effects. Postsynaptic α7nAChR mediate fast inward currents in both inhibitory interneurons and excitatory neurons [37] and can also trigger the activation of Ca^2+^-dependent intracellular signaling pathways such as protein kinase A (PKA), protein kinase C (PKC), extracellular signal-regulated kinase (ERK)1/2, and Ca^2+^/calmodulin-dependent protein kinase II (CaMKII) [36]. Perisynaptic α7nAChRs are located outside the postsynaptic density but can activate intracellular signaling pathways that regulate nearby GABAergic and glutamatergic synapses [38]. Furthermore, activation of α7nAChR can promote long-term potentiation (LTP), the activity-dependent strengthening of synaptic connections considered to underlie memory [39,40]. Consistent with the ability of α7nAChR to modulate neurotransmission and synaptic plasticity in brain areas associated with cognitive functions, α7nAChR has been found to play a role in mediating cholinergic effects on learning, memory, and attention (reviewed by [36]).

## 4. α7nAChR Alterations in AD

α7nAChR is one of the most abundant nAChRs in the mammalian brain, exceeded only by the heteromeric α4β2 receptor [41]. Although distributed widely throughout the brain, the expression of α7nAChR is particularly high in cortical and limbic areas associated with cognitive functioning, including the hippocampus, cortex, and amygdala, as well as in the basal forebrain [42]. Within the brain, α7nAChR is expressed not only by neurons, but by astrocytes [43,44], microglia [45,46], microvascular endothelial cells [47], and NG2-expressing cells [48].

In the post-mortem AD brain, binding of the α7-specific antagonist α-bungarotoxin (α-BTX) is reportedly reduced in hippocampus [49] and temporal cortex [50,51], although not all studies agree [49,52]. In contrast, most studies show no difference in α-BTX binding in the frontal cortex in AD compared to control brains [50,52,53,54]. Similarly, Western blot detection in AD brains has shown a reduction in α7-subunit immunoreactivity in the hippocampus [55] and temporal cortex [56,57] but also in the frontal cortex [58] although again some discrepancies have been reported [55,59].

Detailed immunohistochemical analyses have addressed the question of whether reduced levels of α7nAChR (or α-BTX binding) reflects decreased expression or loss of α7nAChR-expressing cells. Both α7nAChR labeling intensity as well as the number or density of α7-positive neurons were reduced in the hippocampus and temporal cortex [51] and in the superior frontal gyrus [57,60] in AD. In the latter case, it was found that the total number of neurons in the region was not reduced, suggesting lack of expression of α7nAChR rather than outright loss of α7-positive cells. Similarly, Banerjee et al. [61] found that, while the number of α7-expressing neurons in the temporal cortex of AD patients was reduced, the total number of neurons was stable. However, in this study it was found that the remaining α7-positive neurons in the temporal cortex showed particularly intense rather than weak immunolabeling. Interestingly, a disconnect between mRNA and protein expression of α7nAChR in the superior frontal gyrus was reported by Wevers et al. [57,60]. In this case, the density of α7 mRNA+ cells was unchanged in AD, but the density of α7nAChR immunopositive cells was reduced, again arguing against an outright loss of α7nAChR+ cells [57,60]. Hellström-Lindahl et al. [49] reported an increase in α7 mRNA in the hippocampus of AD patients compared to controls but a decrease in α-BTX binding in the same area. These results could reflect an alteration in the translation of mRNA to protein or a failure of assembly or trafficking of the receptor to the cell surface. Alternatively, the discrepancy between mRNA and protein could reflect the fact that the α7nAChR may be expressed on terminals of projection neurons whose cell bodies do not lie in the target area [49]. Counts et al. [62] additionally reported an increase in α7 mRNA in individual cholinergic basal forebrain neurons of patients with mild to moderate AD, although it is unknown whether this translated to increased protein expression. Overall, the results indicate a reduction in α7nACh receptors in the postmortem AD brain. Of note, the early immunohistochemical studies should be interpreted with caution owing to the later finding of the lack of specificity of many commercial α7nAChR antibodies [63,64,65,66].

The recent development of α7nAChR specific positron emission tomography (PET) radioligands has enabled the assessment of α7 levels in the living brain [67,68,69]. Coughlin et al. [70] used 18F-ASEM imaging to quantify α7nAChR receptor availability in patients with mild cognitive impairment (MCI), which in many cases is a prodromal stage of AD. They found an increase in the availability of α7nAChR in multiple brain regions including several cortical regions, hippocampus, and basal forebrain [70]. Together with the post mortem analyses described above, these findings suggest a possible biphasic response in α7nAChR availability, with an initial increase in the prodromal stage and reduction in later stages of disease. However, it would be very informative to track changes in individual patients longitudinally using 18F-ASEM, in relation to neuropathological biomarkers and cognitive status, to better understand the temporal relationship between α7nAChR changes and other pathological alterations in AD. Table 1 summarizes the literature related to α7nAChR changes in the AD brain.

Levels of α7nAChR in transgenic mouse models of AD have also been investigated. In mice expressing human mutant amyloid precursor protein (APP), levels of α7nAChR have been found to increase over time relative to non-transgenic mice [76,78,79,80,81], although in one case, the level of α7nAChR was reduced at 12 months of age despite being elevated at 3, 6, and 9 months of age [81]. In mice co-expressing human mutant APP with a mutant presenilin 1 (PS1; A246E), upregulation of α7nAChR was even more pronounced than in mice expressing mutant APP alone [79]. However, in APP/PS1_G384A_ mice bearing a different PS1 mutation, levels of α7nAChR in brain homogenates were reduced at 1.5 and 6 months of age relative to wildtype controls [82]. Significant reductions in α-BTX binding were also observed in the hippocampus, retrosplenial and parietal cortices, and thalamus of 6-month-old 3xTg mice expressing mutant APP, PS1, and tau [83]. Conversely, no changes in α-BTX binding sites were detected in APPswe/PS1 mice between the ages of 3 weeks and 17 months [84] or in APPswe cortex at 3 months of age [85]. These differences are hard to reconcile but may relate to age-dependent variability in the severity of amyloid pathology or other neuropathological features (e.g., neurofibrillary tangles and neuron loss) between the models.

Given the participation of α7nAChR in mediating cholinergic effects on cognitive functions, it is likely that the loss of cholinergic input to the hippocampus and cortex and/or changes in α7nAChR expression contribute to cognitive deficits in AD. However, evidence suggests that, rather than being an innocent bystander, α7nAChR plays an active role in neuropathological processes as well. α7nAChR is functionally linked to several key pathological players in AD, including Aβ, tau, and neuroinflammation, and these interactions will be discussed in detail in the following sections.

## 5. Physiological and Pathological Effects of Aβ

Aβ peptides are generated by sequential cleavage of APP by β- and γ-secretases. Depending on the exact cleavage site, peptides varying in length from 38 to 49 residues can be produced, with Aβ_1–40_ being the most abundant, followed by Aβ_1–42_ [86,87]. Monomeric Aβ peptides assemble to form soluble oligomers of various sizes and conformations, as well as insoluble fibrils. Fibrils further aggregate, forming amyloid plaques [86]. Although only differing by two amino acids, Aβ_1–40_ and Aβ_1–42_ adopt different conformations and vary considerably in their biophysical properties [86]. Aβ_1–42_ has a higher propensity to aggregate than Aβ_1–40_ [86,88] and is the major species found in amyloid plaques [89,90]. While plaques containing fibrillar Aβ are one of the main pathological features of the AD brain, soluble oligomers are recognized as the more highly pathogenic species and correlate better with measures of cognitive function and neurodegeneration in AD patients than does plaque burden [91,92]. Exogenous treatment with soluble Aβ oligomers alters synapse structure, impairs LTP and facilitates long-term depression (LTD), induces tau hyperphosphorylation, triggers inflammation, promotes cell death, and results in cognitive deficits [93].

In contrast to these damaging effects of exposure to high levels of soluble Aβ, beneficial roles of physiological concentrations of Aβ are increasingly being understood. Aβ has been implicated in protecting against microbial infection, regulating the blood brain barrier, promoting recovery from injury, and regulating synaptic function and thereby cognitive function [94,95,96]. In fact, it has been repeatedly demonstrated that reducing endogenous levels of Aβ by genetic knockout or using β-secretase 1 (BACE1) inhibitors or blocking its effects using antibodies has detrimental effects on cognition and synaptic function [97,98,99,100,101]. Conversely, neurotransmission and synaptic plasticity are enhanced by exposure to a modest increase in oligomeric Aβ, as is the performance of cognitive tasks [96,102,103,104]. The effects of oligomeric Aβ are therefore considered to be hormetic, whereby low concentrations enhance cognition and high concentrations produce detrimental effects [105,106]. This inverted U-shaped dose-response curve can also be seen in the response of various cellular and molecular processes to Aβ [95,105,106,107].

## 6. Aβ Interaction with α7nAChR

A number of cell surface receptors have been identified that participate in various aspects of Aβ function, including prion protein (Prpc), receptor for advanced glycation end products (RAGE), p75 neurotrophin receptor (p75NTR), Nogo-66 Receptor 1 (NgR1), Ephrin type B receptor 2 (EphB2), sortilin, insulin receptor, and α7nAChR, among others (reviewed in [108]). The α7nAChR in particular has been implicated in both the physiological and neuropathological effects of Aβ. In 2000, Wang et al. [109] reported that Aβ_1–42_ and α7nAChR were found in close proximity and could be co-immunoprecipitated from the AD brain. Co-immunoprecipitation of α7nAChR with Aβ from the brains of APPSwe/PS1dE9 transgenic mice and from α7nAChR-expressing HEK293 cells has also been demonstrated [110,111]. Aβ_1–42_ and α7nAChR were capable of forming complexes in vitro, which could be suppressed by treatment with the 12–28 fragment of Aβ, suggesting this sequence contains the binding site for α7nAChR [109]. α-BTX was able to reduce the binding of Aβ_1–42_ to α7nAChR-overexpressing SK-N-MC cells, and Aβ_1–42_ inhibited α-BTX binding to membranes prepared from α7nAChR-overexpressing cells. In addition, overexpression of α7nAChR rendered SK-N-MC cells susceptible to Aβ-induced toxicity [109]. Competitive binding experiments indicated selective, high affinity binding of Aβ_1–42_ to α7nAChR and lower affinity binding to α4β2 nAChR [112]. Using time-resolved fluorescence resonance energy transfer (TR-FRET), Cecon et al. [113] demonstrated saturable and high affinity binding of Aβ_1–42_ oligomers to α7nAChR in HEK293T cells. Oligomer binding could be competed equally by unlabeled oligomeric or monomeric but not fibrillar Aβ, indicating a selectivity for soluble forms of Aβ. Furthermore, Aβ binding was competed by a peptide derived from loop C of α7nAChR which is located near the ligand binding site, suggesting that the Aβ binding site overlaps the orthosteric ligand binding site [113]. In agreement with these findings, in silico docking studies indicated that, within the Aβ_1–42_/α7nAChR complex, the most important interactions occur between residues 12 and 28 of Aβ and loop C of the receptor and the preferential binding site lies in the interface between two α7 subunits [114,115]. Using site-directed mutagenesis of α7nAChR expressed in axonal varicosities of NG108-15 cells, Tong et al. [116] further demonstrated that the Tyr-188 residue (corresponding to Tyr-210 in the proform of the protein) in loop C is critical for the functional response of α7nAChR upon Aβ binding. In addition to this residue, R208 and E211 were predicted by computer-simulated docking to be important for the Aβ_1–42_- α7nAChR interaction [110]. When mutant subunits were created by substituting R208 or E211 for the residues that are found in the analogous positions in the α3 subunit (which does not bind to Aβ), Aβ was unable to be co-immunoprecipitated with the mutant receptors [110]. These results provide a possible explanation for the selectivity of Aβ_1–42_ binding to α4β2 and α7 receptors (in which R208 and E211 are conserved) but not α3β4 receptors [110]. Maatuk and Samson [117] also found that Aβ_1–42_ interacts with the ACh binding site of α7nAChR. Interestingly, modeling studies indicate that α7nAChR might act as a chaperone for the conformational change of Aβ_1–42_ from the α-helical to β-hairpin structure [117]. β-hairpins consist of two antiparallel β-sheets connected by a loop or turn, and it has been suggested that they form the building blocks of toxic Aβ oligomers [118]. If confirmed, this would suggest that interaction with α7nAChR might facilitate the conversion of nontoxic Aβ species to more toxic conformations.

In contrast to the above reports, a number of studies have failed to demonstrate competition of Aβ with α-BTX for binding to α7nAChR [119,120]. In addition, Small et al. [121] found no evidence of Aβ_1–42_ binding to α7nAChR, using several different methods, and instead suggested that Aβ_1–42_ binds to plasma membrane lipids. Aβ interaction with membrane lipids is now well established [122,123], as is the influence of lipids on nAChR function [124]. Interestingly, Aβ/α7nAChR interactions were shown to be modulated by the lipid microenvironment, as cholesterol depletion suppressed the α7nAChR-mediated response to Aβ in presynaptic nerve terminals [125]. Nevertheless, the reason for the discrepancy between the Small et al. [121] study and Wang et al. [109,112] studies regarding Aβ binding to α7nAChR is unclear. Smith et al. [126] also failed to identify α7nAChR as a binding site for oligomeric amyloid in their evaluation of a panel of putative receptors using cell-binding assays in COS-7 and HEK293T cells. However, surface expression of α7nAChR was reportedly low (so low that comparisons could not be made with the rest of the panel of receptors). Although α7nAChR was co-expressed with the chaperone RIC-3, which is essential for assembly of α7nAChR in *Caenorhabditis elegans*, it is not clear whether co-expression with RIC-3 increased the surface expression of α7nAChR in COS-7 or HEK293T cells appreciably. RIC-3 is neither necessary nor sufficient for mammalian α7nAChR assembly; instead, the chaperone NACHO is essential [127]. Therefore, conditions may not have been ideal to detect Aβo binding to α7nAChR in that study. Nevertheless, it should be noted that there are conflicting results regarding the ability of Aβ to bind directly to α7nAChR.

## 7. Aβ Alters Somatodendritic and Presynaptic α7nAChR Activity

The reported high affinity of α7nAChR for Aβ_1–42_ described above suggests that their interaction could occur under normal physiological conditions, and in fact, some effects of Aβ at physiological levels (picomolar concentrations) have been found to be mediated through interactions with α7nAChR. In the AD brain, levels of Aβ_1–42_ reach nanomolar to micromolar levels [128,129] and have detrimental effects. Here, too, the interaction of Aβ with α7nAChR is implicated.

Physiological concentrations of Aβ_1–42_ were shown to elicit α7nAChR-dependent inward currents in Xenopus oocytes [130], although the ability of physiological Aβ concentrations to directly activate α7nAChR is not a consistent finding [121,131,132,133]. Activation of α7nAChR by Aβ_1–42_ was followed by a prolonged period in which the receptor was unable to respond to subsequent application of Aβ_1–42,_ but could still respond to nicotine [130]. In contrast, preincubation with Aβ_1–42_ at high concentrations (between 1 nM and 100 nM) inhibited receptor activation and suppressed nicotine-induced currents in α7nAChR-expressing Xenopus oocytes [130,131,132]. In cultured rat hippocampal neurons as well, nanomolar concentrations of Aβ_1–42_ attenuated the α7nAChR current response to ACh [134]. Similarly, high nanomolar concentrations of Aβ partially blocked carbachol- and ACh-induced currents in hippocampal interneurons and decreased the open channel probability of both α7nAChR and non-α7nAChR [135].

Nicotinic receptors exhibit desensitization upon repeated or prolonged exposure to nicotinic agonists [136], and α7nAChR exhibit particularly rapid desensitization [137]. The inhibitory effects of Aβ on nicotinic currents have been attributed to desensitization, although some research suggests otherwise. Lasala et al. [138] employed single channel recordings and fluorescence spectroscopy using a conformation-sensitive probe to investigate channel activity and conformational changes induced by exposure to oligomeric Aβ in α7nAChR-expressing BOSC23 cells. Low (pM) concentrations of Aβ stimulated α7nAChR channel opening and pushed the receptor towards a desensitized conformation, although the conformation induced by Aβ was distinct from the desensitized state elicited by carbachol. In contrast, high concentrations of Aβ were found to reduce the responsiveness to agonists but did not trigger channel opening. Upon exposure to high concentrations of Aβ, the α7 receptor adopted a stable conformation different from the desensitized state, which the authors interpret as being indicative of a slow open channel block [138]. Therefore, low and high concentrations of oligomeric Aβ appear to drive α7nAChR to different conformational states that determine the response to subsequent application of ligands.

While somatodendritic α7nAChR mediate cholinergic transmission, activation of α7nAChR in the presynaptic compartment of various cell types enables modulation of neurotransmitter release through depolarization of axonal terminals or via stimulation of Ca^2+^-dependent signaling [37]. Physiological concentrations of Aβ_1–42_ evoked sustained increases in Ca^2+^ in isolated presynaptic nerve endings (synaptosomes) from rat brains, and in axonal varicosities of differentiated hybrid neuroblastoma NG108-15 cells, and this was largely inhibited in synaptosomal preparations from mice lacking the α7nAChR subunit or by application of α-BTX [125,139,140]. However, the contribution of α7nAChR relative to non-α7nAChR to Aβ_1–42_ -induced Ca^2+^ responses differed depending on the source of the synaptosomes (hippocampal vs. cortical) and the concentration of Aβ, with higher concentrations seeming to recruit more non-α7 receptors [139,140]. In cultured rat cortical neurons, increasing the extracellular concentration of Aβ, either by inhibiting Aβ degradation or by addition of synthetic Aβ at a physiological concentration (200 pM) induced an increase in synaptic vesicle recycling at both excitatory and inhibitory synapses, while blocking Aβ production had the opposite effect [141]. The effect of Aβ on synaptic vesicle recycling was completely blocked by α-BTX and was dependent on both extracellular Ca^2+^ and Ca^2+^ released from intracellular stores via the ryanodine receptor [141]. In anaesthetized mice, application of low concentrations of monomeric Aβ to hippocampal subfields elicited α7nAChR-dependent glutamate release [142]. Therefore, physiological concentrations of Aβ stimulate presynaptic Ca^2+^ responses at least partially via α7nAChR, leading to increased release of neurotransmitter.

High concentrations of Aβ_1–42_ prevented nicotine-induced Ca^2+^ responses in synaptosomes and in axonal varicosities of NG108-15 cells. However, this was attributed to activation of nAChRs by Aβ, which occludes the response to nicotine [125,140]. Concentrations of Aβ_1–42_ up to 100 nM increased presynaptic Ca^2+^ in axonal varicosities of NG108-15 cells and cortical synaptosomes, an effect which was blocked by α-BTX [125,139] and was dependent on both VGCCs and intracellular Ca^2+^ stores [125]. In contrast, Lazarevic et al. demonstrated that unlike exposure to low picomolar levels of Aβ (40 and 42), which increased synaptic vesicle recycling, low micromolar Aβ (1 μM) decreased synaptic vesicle recycling in cultured cortical neurons. α-BTX partially blocked the effect of 1 uM Aβ_1–42_, suggesting that both α7 and other types of receptors are involved in the inhibitory effect of Aβ on presynaptic neurotransmitter release [141]. The concentration of Aβ used by Lazarevic et al. [141] was quite high (1 μM), and this might explain the inhibitory effect of Aβ on synaptic vesicle recycling in contrast to the facilitatory effect on presynaptic Ca^2+^ which was observed using concentrations up to 100 nM Aβ by Khan et al. and Mehta et al. [125,139]. Notably, although all three doses of Aβ_1–42_ (10 pM, 100 pM, and 100 nM) assessed by Mehta et al. [139] evoked Ca^2+^ responses in cortical synaptosomes, 100 pM Aβ elicited the strongest response, again possibly suggesting a hormetic-like effect on presynaptic α7nAChR.

Taken together, the results may suggest that somatic/dendritic α7nAChR are sensitive to inhibitory effects of Aβ at lower (though still pathological) concentrations, while higher concentrations are necessary to induce inhibitory effects on presynaptic receptors. Khan et al. [125] suggest that the different concentration-dependence of the effects of Aβ on α7nAChR expressed in different neuronal compartments might be due to altered rates of desensitization of receptors in these compartments [125]. The facilitatory effect of Aβ even at relatively high concentrations on presynaptic α7nAChR might be a result of the slow inhibition of presynaptic α7nAChR [125], which in turn might depend on differences in the lipid microenvironment in presynaptic versus somatic/dendritic compartments [125,143]. In support of this idea, disruption of lipid rafts by cholesterol depletion strongly reduced the α7nAChR-dependent presynaptic Ca^2+^ response to Aβ exposure [125].

## 8. Aβ-α7nAChR Interactions Regulate Excitation/Inhibition Balance

The prodromal stage of AD (MCI) is characterized by hippocampal hyperactivity [144]. In mouse models of AD, hippocampal hyperactivity emerges early, even before plaque deposition, suggesting soluble Aβ may be to blame [145,146,147], and this is supported by the finding that extracts of the AD brain containing soluble amyloid, or synthetic dimers, induce neuronal hyperactivity [148]. In addition, AD patients are susceptible to seizures and subclinical epileptiform activity, which may be evident at very early stages [149]. Similarly, mouse models of AD exhibit increased epileptiform activity and seizure susceptibility, reflective of neuronal hyperactivity and excitation/inhibition imbalance leading to network hypersynchrony [150,151,152,153,154,155]. Hyperexcitability is detrimental, resulting in impaired circuit activity and network dysfunction, leading to cognitive impairment [145]. In a longitudinal study, hippocampal hyperexcitability in nondemented patients at baseline was correlated with faster cognitive decline two years later [156]. In addition, hyperexcitability contributes to pathological progression of AD by increasing Aβ levels and stimulating the spread of tau pathology [157]. A number of mechanisms promoting neuronal hyperexcitability in AD have been described, involving both increased excitatory transmission and reduction in inhibitory control [157].

Evidence suggests that under normal conditions α7nAChR helps to regulate both excitation and inhibition in hippocampal and cortical circuits. α7nAChR can potentiate excitatory transmission by promoting presynaptic glutamate release and/or through postsynaptic mechanisms on the target cell [35,158]. In addition, cholinergic input, acting through α7nAChR, influences excitatory neurons by altering the activity of GABAergic interneurons that synapse either directly on the excitatory cell or on a second inhibitory interneuron which synapses on the excitatory cell. This enables both direct inhibition and indirect disinhibition of pyramidal neurons [32,159,160,161].

Excitatory and inhibitory neurons are differentially affected by Aβ, and this may be mediated at least in part by α7nAChR. Hippocampal interneurons express high levels of α7nAChR, while low levels of α7nAChR are found in pyramidal neurons [162,163]. Furthermore, the pattern of distribution is distinct, with inhibitory interneurons expressing somatic/dendritic and presynaptic α7nAChR [164,165,166], while excitatory pyramidal neurons express mainly presynaptic α7nAChR [39,164,165,167]. In cultured mouse hippocampal neurons, exposure to high concentrations of oligomeric Aβ_1–42_ (250 nM and 500 nM) increased neuronal Ca^2+^ activity [164]. However, using cell-type specific expression of a Ca^2+^ indicator, it was shown that Ca^2+^ activity was elevated only in excitatory neurons while GABAergic interneurons exhibited reduced Ca^2+^ activity. Treatment with the GABA_A_ receptor agonist muscimol prevented the Aβ-induced Ca^2+^ hyperactivity in excitatory neurons, implicating a reduction in GABAergic inhibitory signaling in generating the hyperactivity observed in excitatory cells. The effects of Aβ could be mimicked by coapplication of α7 and α4β2 antagonists, but not by either alone, suggesting inhibition of both receptors by Aβ may be required to induce Ca^2+^ hyperactivity [164]. The authors suggest that Aβ_1–42_ inhibits α7nAChR and α4β2 activity in inhibitory interneurons, reducing the inhibitory input to excitatory pyramidal neurons, leading to hyperexcitability [164].

Interestingly, blocking α7nAChR in the CA1 area of the hippocampus using methyllycaconitine (MLA) has been shown to increase the excitability of pyramidal neurons and the susceptibility to pilocarpine-induced seizures; this was attributed to decreased activity of interneurons leading to disinhibition of pyramidal neurons [163]. Within CA1, α7nAChR was found to be mainly located in parvalbumin+ interneurons, suggesting that inhibition of these cells by MLA was likely responsible for the increased excitability of pyramidal cells. Hippocampal parvalbumin+ interneurons are reduced in human AD brains and in some animal models [168,169], and dysfunction of parvalbumin interneuron-mediated inhibition contributes to excitation/inhibition imbalance and altered oscillatory activity in the AD hippocampus [169]. The role of the Aβ-α7nAChR interaction specifically in interneuron populations (such as the parvalbumin-positive population) in generating hyperexcitability therefore warrants further investigation.

Liu et al. [170] found that exposure to 100 nM fibrillar, and to a lesser extent, oligomeric, Aβ resulted in hyperexcitability of pyramidal neurons in mouse primary hippocampal culture and hippocampal slice culture, although this was only observed after treatment with Aβ for 7 or 10 days but not after shorter exposures (up to 4 days). The frequency of spontaneous AMPA-receptor-mediated miniature excitatory postsynaptic currents (mEPSCs) was increased by chronic Aβ exposure as well, suggesting an increase in presynaptic vesicular glutamate release. The effects of Aβ were abolished in cultures from α7-subunit-knockout mice or by continuous treatment with MLA, and even acute inhibition of α7nAChR by MLA in cultures pre-exposed to Aβ inhibited the hyperexcitation [170]. Remarkably, Aβ-induced hyperexcitation was preceded by an upregulation of α7nAChR expressed at the cell surface, as well as an increased current response to the α7 selective agonist choline in pyramidal neurons. Conversely, GABAergic neurons did not exhibit increased α7nAChR current responses. Therefore, an increase in surface α7nAChR expression might contribute to the hyperexcitability of pyramidal neurons after chronic exposure to Aβ [170]. While both Sun et al. [164] and Liu et al. [170] demonstrated α7nAChR-dependent hyperexcitation in response to Aβ, the different time course of the effects suggests that the mechanisms underlying hyperexcitation in these two studies may not be the same. Whether inhibitory interneurons contributed to producing pyramidal neuron hyperexcitability was not explored in this study but would be of great interest.

**Figure 1:**
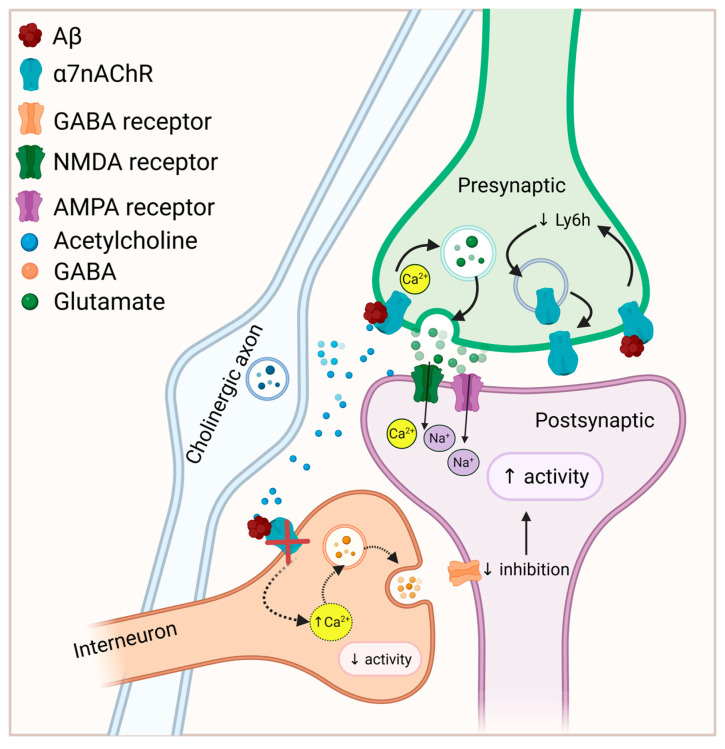
Potential mechanisms linking Aβ-α7nAChR interaction to neuronal hyperexcitability. Depending on the concentration, Aβ-α7nAChR interaction may facilitate presynaptic glutamate release. Aβ inhibits α7nAChR (and α4β2) activity in GABAergic interneurons, decreasing inhibitory input to excitatory pyramidal neurons, resulting in hyperexcitability. In addition, Aβ binding to α7nAChR promotes α7nAChR assembly and surface expression by decreasing Ly6h, which under normal conditions reduces receptor assembly. While increased α7nAChR surface expression has been associated with hyperexcitability, the mechanisms mediating this effect are unknown. Solid arrows represent processes that may be augmented by Aβ binding while dashed arrows represent processes that are attenuated by Aβ binding. Figure created in https://BioRender.com.

In the above study, the increased cell surface expression of α7nAChR in response to chronic Aβ was not accompanied by increased total α7nAChR protein or mRNA and was prevented by an inhibitor of protein trafficking, suggesting that chronic exposure to Aβ stimulated the assembly or translocation of α7nAChR to the cell surface [170]. Elevated cell surface expression and/or function of α7nAChR in response to long-term (at least 24 h) Aβ exposure has also been reported in differentiated SH-SY5Y cells [171], PC12 cells [172], and in cultured hippocampal slices [80]. A mechanism for enhanced α7nAChR surface expression in response to chronic Aβ exposure was proposed by Wu et al. [173]. They found that the assembly of α7nAChR homomers in hippocampal pyramidal neurons was dually modulated by two proteins with opposing effects: NACHO enhances, while Ly6h reduces, α7nAChR assembly. Exposure to a fibril-enriched preparation of Aβ for 7 days led to a downregulation of Ly6h, which was blocked by MLA. By downregulating Ly6h, the balance would tip towards α7nAChR assembly, leading to α7nAChR upregulation. Thus it appears that Aβ acts through α7nAChR to upregulate α7nAChR surface expression via downregulation of Ly6h [173], and this may lead to neuronal hyperexcitability [170]. Figure 1 illustrates how Aβ-α7nAChR interaction may lead to excitation/inhibition imbalance.

In the AD brain, early hyperexcitability is followed eventually by hypofunction at later stages of the disease [157,174]. In cholinergic neurons from Aβ_1–42_-expressing *Drosophila melanogaster*, spontaneous activity was increased relative to controls at early timepoints (up to 5 days in vitro), and this was likely a presynaptic effect since the frequency of mEPSCs was increased without a change in other mEPSC parameters [175]. Similarly, application of exogenous Aβ_1–42_ to wildtype neurons also increased the frequency of spontaneous EPSCs. In neurons from the Drosophila α7 (Dα7)-null mutant line, the frequency of mEPSCs was unaltered in response to Aβ treatment, implicating α7 in Aβ-induced neuronal hyperactivity. In contrast to early hyperexcitability, neurons expressing Aβ_1–42_ or exposed to exogenous Aβ_1–42_ showed reduced frequency of mEPSCs after several days. Notably, even a relatively short exposure to exogenous Aβ_1–42_ (up to an hour) led to synaptic silencing in wildtype neurons days later but not in neurons from Drosophila lacking Dα7 [175]. Furthermore, inhibiting the early enhanced synaptic activity in Aβ_1–42_-expressing neurons using curare prevented the synaptic inhibition at later timepoints. The authors suggest that increased activity caused by exposure to Aβ_1–42_ might initiate α7nAChR-dependent homeostatic mechanisms that eventually result in synaptic inhibition [175]. The mechanisms mediating the link between α7nAChR-dependent activity and synaptic inhibition have not been elucidated. Furthermore, whether and how this effect, which occurs over days in vitro, might contribute to the emergence of hypofunction in AD brains is unknown.

## 9. α7nAChR Mediates Aβ Effects on Synaptic Plasticity

In addition to its role in regulating neurotransmission and excitation/inhibition balance, α7nAChR has been implicated in regulating synaptic plasticity and in mediating the effects of both physiological and pathological levels of Aβ on plasticity. Depending on the timing of cholinergic activation relative to other inputs and the location of the receptors, α7nAChR can promote LTP or LTD and convert short-term potentiation to either LTP or short-term depression (reviewed in [32]). LTP at Schaffer collateral-CA1 synapses in hippocampal slices is facilitated by physiological concentrations of Aβ (with peak effect at 200 pM) but not in slices from α7-knockout mice [102]. Similarly, conversion of early (E)-LTP to late (L)-LTP in hippocampal slice cultures was facilitated by exposure to 200 pM oligomeric Aβ_1–42_, but Aβ was incapable of converting E-LTP to L-LTP in hippocampal slices from α7-subunit-knockout mice or when slices from normal mice were treated with MLA [103]. Furthermore, α7 gene deletion prevented the ability of intrahippocampal injection of 200 pM Aβ_1–42_ to prolong the retention of novel object memory and promote both reference memory in the Morris Water Maze and contextual fear conditioning [102,103]. α7nAChR therefore mediates the effects of physiological Aβ on synaptic plasticity, which may in turn modulate cognitive functions.

In contrast, high concentrations of oligomeric Aβ_1–42_ impair hippocampal LTP [40,102], and Aβ-induced deficits in LTP can be rescued by treatment with α7nAChR agonists [176,177,178]. In addition, MLA was able to inhibit LTP suppression elicited by an Aβ peptide fragment, suggesting that α7nAChRs participate in Aβ-induced LTP impairment [179]. Furthermore, Aβ induces protein phosphatase 2B (PP2B/calcineurin)-dependent endocytosis of N-methyl-D-aspartate (NMDA) receptors through its interactions with α7nAChR [180], which could impair synaptic plasticity.

## 10. α7nAChR and Aβ-Induced Neurotoxicity

At pathological concentrations Aβ is toxic to neurons, and evidence indicates that α7nAChR may be involved in modulating Aβ-induced toxicity. Studies demonstrating the neuroprotective effects of α7 antagonists and α7-subunit gene deletion against Aβ-induced neurotoxicity in primary neuron cultures and differentiated SH-SY5Y cells suggest that the Aβ-α7nAChR interaction contributes to neurotoxicity [181,182]. Notably, the Aβ-induced α7nAChR upregulation and neuronal hyperexcitation observed by Liu et al. [170] were associated with reduced neuron viability [170,171] indicating that increased α7nAChR expression may contribute to Aβ-induced neurotoxicity. Furthermore, elevated α7 expression in response to Ly6h knockdown increased basal rates of neuron death and potentiated neurotoxicity induced by H_2_O_2_ or hypoxia/glucose deprivation in cultured hippocampal neurons [173]. In contrast, knockdown of NACHO, which reduced α7nAChR expression, had the opposite effects [173]. Together these results suggest that increased signaling through α7nAChR via receptor upregulation may contribute to Aβ-induced neurotoxicity and additionally increase the susceptibility of neurons to other insults [173]. In line with this, previous work demonstrated that a gain of function mutation in α7nAChR and exposure to an α7nAChR positive allosteric modulator were cytotoxic [183,184] supporting the notion that excessive α7nAChR activity can be harmful.

Conversely, other studies have found that the toxicity of Aβ was exacerbated by α7 silencing in undifferentiated SH-SY5Y cells [185,186] and by α-BTX treatment in both nerve growth factor (NGF)-differentiated PC12 and undifferentiated SH-SY5Y cells [172,186]. Whether this might relate to differences between primary culture and cell lines, or some other experimental factor is not known. Undifferentiated SH-SY5Y cells are considered to be immature catecholaminergic neurons [187] while NGF-differentiated PC12 cells are sympathetic nerve-like cells [188] and may display important differences in α7nAChR function compared to primary neurons. In addition, the interplay between excitatory and inhibitory cells present in primary culture is lacking in immortalized cell lines.

α7nAChR agonists have frequently been shown to be neuroprotective against toxicity induced by Aβ or other insults relevant to AD pathological processes (glutamate toxicity, oxidative stress) through modulation of Janus kinase 2 (JAK2)/signal transducer and activator of transcription 3 (STAT3), phosphoinositide 3-kinase (PI3K)/Akt, and mitogen-activated protein kinase (MAPK) pathways (reviewed by [189,190]). Interestingly, chronic exposure to nicotine and other nicotinic agonists also increases the cell surface expression of nAChRs, including α7nAChR [191,192], and the neuroprotective efficacy of different agonists is correlated with their ability to upregulate α7nAChR [193], suggesting that upregulated α7nAChR is not always detrimental. However, nicotine and other α7nAChR agonists stimulate neuroprotective pathways that likely differ from pathways stimulated by Aβ which might explain their neuroprotective ability despite inducing α7nAChR upregulation. Clearly, the role of α7nChR in mediating or mitigating the neurotoxic effects of Aβ requires further investigation.

## 11. α7nAChR and Intraneuronal Amyloid

Aβ-induced neurotoxicity is particularly associated with intraneuronal localization of the peptide [194,195]. In the human AD brain, as well as in the brains of many AD mouse models, intracellular Aβ accumulation has been detected within vulnerable neuron populations and precedes the formation of neurofibrillary tangles and extracellular plaques [195,196]. Intraneuronal amyloid can derive from intracellularly produced Aβ and/or the internalization of extracellular Aβ, although the extent to which each of these mechanisms contributes to the accumulation of Aβ within the cells, and whether their downstream consequences are equivalent, is not clear [197,198]. Furthermore, there is evidence that the uptake of extracellular Aβ_1–42_ enhances the production and accumulation of intracellular Aβ, further amplifying the effects of Aβ_1–42_ uptake [197,199,200,201]. The appearance of intraneuronal amyloid is associated with synaptic deficits, neuron loss, neuroinflammation, and cognitive impairments [196,202,203,204,205].

Amyloid uptake by neurons may be facilitated by the Aβ-α7nAChR interaction. In the postmortem AD brain, α7nAChR and Aβ_1–42_ were found to be localized in the same neurons, and neurons bearing high levels of Aβ_1–42_ exhibited intense α7nAChR immunostaining [206]. SK-N-MC neuroblastoma cells overexpressing α7nAChR showed much greater intracellular accumulation of exogenously applied Aβ_1–42_ than control cells. Surface expression of α7nAChR was depleted upon Aβ treatment and appeared to redistribute to intracellular deposits, where it colocalized with Aβ_1–42_. Treatment with either α-BTX or an endocytosis inhibitor prevented intracellular Aβ accumulation as well as the redistribution of α7nAChR [206]. SH-SY5Y cells were also shown to internalize Aβ_1–42_ oligomers, which then co-localized with α7nAChR within the cells, while MLA decreased levels of intracellular Aβ, and treatment with the α7 agonist PNU 282987 promoted internalization [207]. However, in this study the ELISA measurements of cytosolic amyloid indicated very small changes in Aβ content upon exposure to exogenous Aβ, suggesting quite low levels of amyloid uptake relative to the level of endogenous intracellular Aβ.

In contrast to the above studies, Saavedra et al. [208] failed to find an effect of α-BTX on Aβ internalization by distal axons of basal forebrain cholinergic neurons, even though α7nAChR is enriched in the distal axons of these cells and the cells did take up Aβ. Of note, in this study, apolipoprotein E (ApoE) was deliberately excluded from the cultures. ApoE is a lipoprotein that acts as a chaperone for Aβ and has been shown to enhance the uptake of soluble Aβ into synaptic terminals [209]. Furthermore, ApoE interacts with α7nAChR [210] so the omission of ApoE might have affected α7nAChR-dependent Aβ internalization.

Although intraneuronal Aβ is considered detrimental, research by Hung et al. [186] suggested that internalization of Aβ/α7nAChR complexes promoted autophagic processing of Aβ, thereby limiting its neurotoxicity. Nevertheless, neurons expressing α7nAChR appear to be highly vulnerable in AD, and this may relate to their susceptibility to accumulation of intraneuronal amyloid [211].

## 12. Relationship of α7nAChR to Tau Phosphorylation

In the AD brain, tau becomes hyperphosphorylated, leading to misfolding and aggregation into neurofibrillary tangles [212], and there is a complex bidirectional interplay between Aβ and tau [213]. Signaling through the α7nAChR may provide a link between Aβ and tau phosphorylation, since it was found that α7nAChR ligands and antisense oligonucleotides are capable of inhibiting tau phosphorylation induced by Aβ_1–42_ [214,215]. The signaling mechanism linking Aβ-α7nAChR interaction with tau hyperphosphorylation might include MAP kinase-dependent tau kinase activation. Exposure to Aβ_1–42_, at doses ranging from 100 pM to 100 nM, leads to a α7nAChR-dependent phosphorylation and activation of extracellular signal-regulated kinase 2 (ERK2) through PI3K in hippocampal slices and in SH-SY5Y cells [80,216,217]. Activation of ERK1/2 in AD models is associated with increased tau phosphorylation via stimulation of glycogen synthase kinase 3 (GSK-3) activity [218]. In particular, phosphorylation of GSK-3β at the Tyr216 residue is associated with stimulation of tau phosphorylation [219]. Hu et al. [215] found that Aβ-induced GSK-3β phosphorylation at Tyr216 in PC12 cells was partially inhibited by MLA, α-BTX, and the α7nAChR agonist A-582941, implicating α7nAChR in Aβ-induced tau phosphorylation by GSK-3β.

## 13. Heteromeric α7β2 Receptors

While much of the research into the role of α7nAChR in AD has focused on homomeric α7 receptors, recent work provides evidence for the existence of α7β2 heteromeric receptors and implicates these receptors in neuropathological processes. α7 and β2 subunits colocalize in basal forebrain cholinergic neurons [220], and when expressed heterologously in Xenopus ooctyes, they are capable of forming functional nAChRs with kinetic and pharmacological properties that are distinct from α7 homomeric receptors [221]. Naturally occurring α7β2 receptors were later described by Liu et al. [222] in rodent basal forebrain cholinergic neurons. Choline-induced responses by these receptors were highly sensitive to oligomeric Aβ-induced suppression but were much less affected by fibrillar or monomeric Aβ [222]. In Xenopus oocytes, heterologously expressed α7β2 nAChRs were sensitive to the effects of low nanomolar concentrations of Aβ on choline-induced currents; using the same concentration of Aβ, homomeric α7 receptors were not affected [222]. α7β2 receptors have since been demonstrated in mouse hippocampal CA1 GABAergic interneurons (which were also found to be highly sensitive to oligomeric Aβ) and in the human brain [223,224,225].

Oligomeric Aβ_1–42_ activated both α7 and α7β2 nAChR in transfected SH-EP1 cells but enhanced single-channel open-dwell times preferentially in α7β2 receptors [226]. Medial septum/diagonal band and horizontal band neurons (but not nucleus basalis neurons) in basal forebrain organotypic slice cultures exposed to oligomeric Aβ_1–42_ for 9 days exhibited hyperexcitation which was normalized by coapplication of MLA and in slices obtained from β2 subunit knockout mice. The ability of β2 deletion to mimic the effects of MLA (which antagonizes both homomeric α7 and α7β2 receptors) suggests that the effects of Aβ are dependent on α7β2 receptors [226]. The expression of α7β2 receptors is restricted to certain neuronal populations, and given the sensitivity of α7β2 nAChRs to Aβ, it has been hypothesized that they might contribute to the selective vulnerability of these cells [226].

Since α-BTX and MLA antagonize both α7 homomeric and α7β2 nAChRs, it is possible that some effects that have been attributed to α7 homomeric receptors might actually be a result of α7β2 nAChR. Recently, analogs of α-conotoxin PnIC that act as selective ligands for α7β2 receptors were discovered [227]; these will be highly valuable in parsing out the functions of homomeric and heteromeric α7-containing receptors in the response to Aβ, both physiological and pathological.

## 14. Astrocytic α7nAChR in AD

α7nAChR is expressed not only by neurons, but also by both astrocytes and microglia in the brain [43,44,45,46], and evidence suggests that glial α7nAChR mediate some of the effects of Aβ in these cell types and may contribute to neuropathological processes. Conversely, cholinergic input acting through glial α7nAChR may play a protective role against AD-associated processes.

Astrocytes carry out a myriad of metabolic, homeostatic, and neuroprotective functions in the normal brain. They contribute to the development, maturation and fine-tuning of neuronal circuits, maintain the spatial and temporal fidelity of synaptic transmission by taking up excess neurotransmitter, and release gliotransmitters in response to integrated synaptic and neuromodulatory information, thereby tuning neuronal activity in response to brain states (e.g., arousal and wakefulness). In addition, astrocytes promote the survival of neurons and participate in inflammatory responses in the brain [228,229,230,231].

In AD, astrocytes in the vicinity of amyloid plaques become hypertrophic, cluster around amyloid plaques, and take up Aβ. This may serve a protective function by forming a barrier around plaques, containing toxic oligomers and limiting their spread, and by phagocytosing dystrophic neurites [232,233]. However, astrocytes in the AD brain take on an inflammatory phenotype, releasing cytokines which contribute to neuronal damage [234].

Some astrocytes in human brain express α7nAChR [74], and astrocytic expression of α7nAChR was found to be increased in the hippocampus, subiculum, entorhinal cortex, and temporal cortex of the AD brain [51,74,75,77]. Interestingly, astrocytes expressing α7nAChR were spatially associated with neuritic plaques, and there was a positive correlation between the percentage of astrocytes expressing α7 and both the number of neuritic plaques in the hippocampus and temporal cortex and the density of neurofibrillary tangles in CA1 [51]. In contrast, in the same study, the number of α7 positive neurons and the intensity of immunolabeling for α7 in neurons was found to be reduced in AD brains compared to controls [51].

Interestingly, exposure of cultured rat primary astrocytes to aggregated Aβ_1–42_ (0.1–100 nM, but not 1 μM) for 48 h resulted in elevated α7nAChR mRNA and protein levels [235] suggesting the increased expression of α7nAChR in astrocytes in the AD brain may be a response to Aβ exposure.

Astrocytic Ca^2+^ signaling mediates the interaction between astrocytes and neurons at local synaptic sites and across wider areas to modulate network activity through the release of gliotransmitters [236,237,238]. Astrocytes are responsive to cholinergic input, which contributes to astrocyte modulation of synaptic transmission and plasticity [239,240,241]. While these functions are known to be mediated by both muscarinic and nicotinic receptors located on astrocytes [240,242,243,244], relatively little is known about the role of α7nAChR specifically. Exposure to nicotine or ACh evokes an α7nAChR-dependent increase in intracellular Ca^2+^ in astrocytes [43,235,245], and activation of α7nAChR triggers the release of glutamate from hippocampal and cortical gliosomes [246,247]. Astrocytes can also modulate neuronal activity through the secretion of factors that affect the expression or distribution of synaptic receptors [248,249,250,251,252], and some evidence suggests α7nAChR may participate in this process [253]. Through these actions, astrocytic α7nAChR play a role in cognitive functions. For instance, astrocytic α7nAChR-stimulated glutamate release has been shown to regulate inhibitory control of dentate granule cells in the hippocampus via activation of inhibitory hilar interneurons, which may contribute to the prioritization of encoding versus memory retrieval in response to cholinergic input from the basal forebrain [254,255]. Furthermore, the critical role of astrocytic α7nAChR in the persistence of memory for the association of an auditory stimulus with footshock was recently demonstrated by Zhang et al. [256].

The function of astrocytic α7nAChR is modulated by Aβ. Exposure of mouse primary astrocytes to physiological (pM) levels of a predominantly monomeric preparation of Aβ_1–42_ increased the frequency and amplitude of Ca^2+^ transients [257]. The effect of Aβ_1–42_ was dependent on α7nAChR, since the effect was abolished after treatment with MLA and in astrocytes harvested from α7 knockout mice [257]. In rat hippocampal slices, application of a physiological concentration (300 pM) of an oligomeric preparation of Aβ_1–42_ increased the frequency of astrocytic Ca^2+^ transients; MLA inhibited the effect of Aβ_1–42_, while the positive allosteric modulator PNU120596 enhanced the response [258]. These results suggest that the astrocytic Aβ_1–42_ -α7nAChR interaction might serve a physiological role in modulating neuron–glia signaling [257].

Exposure to a pathologically relevant concentration of Aβ_1–42_ (10 nM) also increased astrocytic Ca^2+^ transients in rat hippocampal slices through α7nAChR [258]. Notably, this dose of oligomeric Aβ_1–42_ also increased the frequency of slow inward currents (SICs) recorded from CA1 pyramidal neurons which was blocked by either MLA or the NMDA receptor antagonist D-APV. SICs are generated by activation of extrasynaptic NMDA receptors (eNMDARs) in response to astrocytic glutamate release and serve to modulate neural excitability. Thus, Aβ_1–42_, acting through α7nAChR, can stimulate astrocytes to release glutamate, which can then regulate neuronal activity [258]. Furthermore, hippocampal slices from Tg2576 mice exhibited elevated spontaneous astrocytic activity compared to the wildtype, along with increased SIC frequency and altered responses to an α7nAChR agonist, indicating that chronic exposure to Aβ dysregulates α7nAChR-dependent astrocyte activity [258].

**Figure 2:**
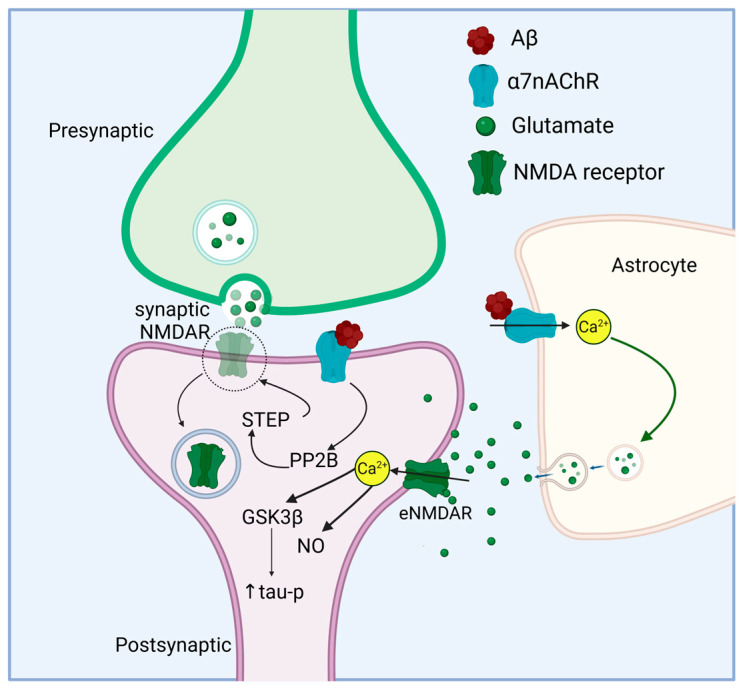
Aβ acts through neuronal and astrocytic α7nAChR to affect NMDA receptor activity. Aβ interaction with neuronal α7nAChR leads to NMDA receptor internalization by activating PP2B, which dephosphorylates and activates STEP (striatal-enriched phosphatase). STEP dephosphorylates the NMDA receptor, triggering endocytosis [180]. In addition, Aβ increases astrocytic intracellular Ca^2+^ through α7nAChR, which stimulates glutamate release. Glutamate released from astrocytes acts on extrasynaptic NMDA receptors leading to tau hyperphosphorylation via GSK3β activation and NO signaling. Activation of extrasynaptic NMDA receptors and reduction in synaptic NMDA receptors may cause a shift from pro-survival to pro-death signaling. Figure created in https://BioRender.com.

Talantova et al. [259] also demonstrated an effect of astrocytic Aβ-α7nAChR interaction on gliotransmission. They found that exposure to Aβ from the human postmortem AD brain or oligomerized synthetic Aβ resulted in α7nAChR-dependent local increases in glutamate in both mixed neuronal/astrocytic and pure astrocytic cultures. In addition, exposure to Aβ elicited a tonic inward current in autaptic neuron cultures, which represents activation of extrasynaptic glutamate receptors by astrocytic release of glutamate. eNMDARs are located away from the postsynaptic density and are not activated during low frequency synaptic events [260]. They are important in generating long-term depression (LTD) under physiological conditions but drive excitotoxicity and pro-death processes under pathological conditions [261,262]. Activation of eNMDARs in response to Aβ resulted in elevated intracellular Ca^2+^ levels in neurons; this was abrogated in cultures depleted of astrocytes. In contrast, mEPSCs, reflecting synaptic currents, decreased in frequency upon Aβ exposure, and this was also dependent on the presence of astrocytes in the culture. The reduction in mEPSCs was attributed to synaptic injury resulting from activation of eNMDARs, elevated neuronal Ca^2+^ levels, and downstream nitric oxide (NO) activation and tau phosphorylation [259]. Activation of extrasynaptic NMDARs by excessive glutamate released from astrocytes upon Aβ/α7nAChR interaction might therefore contribute to hyperexcitation and neuropathology in AD. The role of α7nChR in mediating the effects of Aβ on synaptic and extrasynaptic NMDA receptors is illustrated in Figure 2.

In addition to regulating gliotransmission, cholinergic signaling through α7nAChR contributes to the anti-inflammatory and anti-oxidative function of astrocytes. Cholinergic agonists, acting through α7nAChR, have been shown to blunt the release of cytokines in response to pro-inflammatory lipopolysaccharide (LPS) treatment in astrocytes [263,264,265,266] as well as a variety of other insults (interleukin [IL])-1β exposure [266]; H_2_O_2_ [181], 1-methyl-4-phenylpyridinium (MPP+) [263]). A number of mechanisms have been implicated in the α7nAChR-mediated anti-inflammatory astrocytic response, including regulation of LPS-induced Erk1/2 and p38 MAPK phosphorylation [263]; prevention of nuclear translocation of nuclear factor-kappa B (NF-κB) by inhibiting NF-κB inhibitor alpha (IkBa) phosphorylation [265]; and upregulation of nuclear factor erythroid 2–related factor 2 (Nrf-2) responsive anti-oxidant genes such as heme oxygenase I (HO1), thioredoxin reductase 1 (TXNRD1), and glutamate-cysteine ligase catalytic subunit (GCLC) [265]. Furthermore, while conditioned media from LPS-stimulated astrocytes induced caspase 3/7 activation in neurons, this effect was attenuated when neurons were treated with conditioned media from astrocytes exposed to LPS and the α7nAChR agonist GTS21 [265]. Astrocytes treated with α7nAChR agonist PNU282987 also protected co-cultured neurons from apoptosis induced by exposure to the neurotoxic anti-cancer drug oxaliplatin [267]. Thus α7nAChR contributes to the neuroprotective function of astrocytes.

Aβ induces oxidative stress and pro-inflammatory responses in astrocytes [268,269,270]. However, the role of α7nAChR in either contributing to this response, or in protecting against it, has hardly been investigated. Astrocytes exposed to Aβ_1–42_ released increased levels of macrophage inflammatory protein-1 alpha (MIP-1α), regulated on activation, normal T-cell expressed and secreted (RANTES), IL-1β, IL-6, and tumor necrosis factor α (TNF-α), while pre-treatment with nicotine attenuated the secretion of pro-inflammatory mediators [271]. The authors attribute this to activation of α7nAChR, since α7nAChR expression was confirmed in astrocytes. However, astrocytes also express other nicotinic receptors [242] which might have contributed to the effect of nicotine. In another study, application of Aβ_1–40_ or Aβ_25–35_ in hippocampal astrocytes increased reactive oxygen species (ROS) production via a Ca^2+^-independent mechanism involving nicotinamide adenine dinucleotide phosphate (NADPH) oxidase; Aβ_25–35_ induced caspase 3 in astrocytes as well. Treatment with an anti-α7 antibody reduced Aβ-stimulated ROS production and caspase 3 activation as did treatment with nicotine and α-BTX [272], suggesting that α7nAChR may play a role in these Aβ-induced effects. While the contribution of α7nAChR to the astrocytic response to Aβ has not been thoroughly studied, the ability of α7nAChR ligands to modulate the astrocytic response to other inflammatory stimuli suggests that targeting α7nAChR might be beneficial in AD.

## 15. Microglial α7nAChR in AD

Microglia function as innate immune cells in the brain and participate in surveillance, injury repair, phagocytosis of apoptotic cells, and engulfment of damaged synapses [273,274]. They become activated in response to injury, disease, or infection and coordinate the neuroinflammatory response to these insults [273,275].

In the periphery, signaling from the efferent vagus nerve protects against systemic inflammation via a mechanism known as the cholinergic anti-inflammatory pathway (CAP) which involves cholinergic modulation of pro-inflammatory cytokine release from macrophages [276]. α7nAChR is a critical component of the CAP [277]. Shytle et al. [45] questioned whether a similar pathway involving microglia as the brain-resident macrophage population might exist in the brain. They demonstrated that, primary mouse microglia, immortalized mouse N9 microglia and microglia in mouse brain sections expressed α7nAChR. In primary mouse microglia, pre-treatment with either nicotine or ACh attenuated LPS-induced TNF-α release as well as p38 MAPK and p44/42 MAPK phosphorylation, suggesting an anti-inflammatory effect of cholinergic signaling in the brain. The effects of ACh/nicotine were prevented by the addition of α-BTX, implicating α7nAChR [45]. Similarly, LPS-induced TNF-α release was inhibited in an α7nAChR-dependent manner by nicotine in primary rat microglia [278]. Interestingly, Suzuki et al. [46] demonstrated that, while exposure to nicotine inhibited LPS-stimulated TNF-α release mediated via TLR4, it enhanced TNF-α release mediated by the purinergic P2X7 receptor (P2X7R) in response to adenosine triphosphate (ATP), and in both cases, the effects of nicotine were reversed by MLA. ATP-stimulated TNF-α release is of much smaller amplitude than that induced by LPS and serves a protective role; thus α7nAChR may suppress inflammatory signaling in response to LPS and enhance protective effects in microglia treated with ATP [46]. ACh has been shown to inhibit the LPS-stimulated release of IL-1β and IL-6 from BV2 microglia and increase levels of the anti-inflammatory cytokines IL-4 and IL-10 as well as Arg-1. In addition, ACh inhibited p38 MAPK phosphorylation and prevented the suppression of JAK2/STAT3 and PI3K-Akt activation induced by LPS [279]. Knockdown of α7nAChR expression using short hairpin RNA (shRNA) abrogated the effects of ACh on LPS-induced changes.

Of note, (in [46]) nicotine-induced currents were not detected in microglia, suggesting a non-ionotropic mode of action of α7nAChR in microglia. In fact, it was found that, while nicotine enhanced intracellular Ca^2+^ levels, it was able to do so even when Ca^2+^ was omitted from the extracellular medium, but not when intracellular Ca^2+^ stores were depleted nor in the presence of an intracellular Ca^2+^ chelator, a phospholipase C (PLC) inhibitor, or an inositol triphosphate (IP3) receptor antagonist. These results all suggest that α7nAChR may act mainly as a metabotropic receptor in microglia, signaling through the PLC/IP3 pathway to release Ca^2+^ from intracellular stores [46]. An interaction between α7nAChR and G proteins in the EOC20 microglial cell line has been confirmed [280]. α7nAChR could be co-immunoprecipitated with G proteins (mainly G_αi_), and inhibition of either G_αi_ or PLC reduced the intracellular Ca^2+^ response to choline, as did preincubation with α-BTX. Choline inhibited p38 MAPK activation and TNF-α release induced by LPS exposure, and these effects were mediated by α7nAChR signaling through Gαi and intracellular Ca^2+^ release via the IP3 receptor [280].

An additional protective mechanism mediated by α7nAChR in microglia was reported by Morioka et al. [281,282]. They found that nicotine treatment, acting through α7nAChR, increased the expression of the glutamate/aspartate transporter (GLAST) in cortical microglia, which led to an increase in glutamate uptake. The signaling pathway linking α7nAChR activation to increased GLAST expression involves IP3-induced Ca^2+^ release from intracellular stores, CaMKII activation, and elevated fibroblast growth factor-2 expression [281,282,283]. Clearance of glutamate from synapses by microglial glutamate transporters may serve to limit excitotoxic damage associated with a number of pathological conditions [283], and these results suggest that cholinergic signaling, via α7nAChR, might enhance this process.

As described above, signaling through α7nAChR regulates neuroinflammation and modulates microglial responses to LPS, a canonical PAMP (pathogen-associated molecular pattern). While LPS and Aβ may stimulate some common inflammatory pathways (see [284,285,286,287] they also act through distinct pathways. For example, Zhu et al. [288] demonstrated that LPS upregulated components of a resolution pathway as well as α7nAChR, but exposure to Aβ_1–42_ did not have the same effect and in fact decreased the expression of α7nAChR. Only a handful of studies have specifically examined the role of microglial α7nAChR in modulating neuroinflammatory responses induced by exposure to Aβ. Nicotine, acting through α7nAChR, inhibited ROS production stimulated by fibrillar Aβ_1–42_ in primary rat microglia as well as the translocation of NADPH oxidase subunits from the cytosol to the cell membrane and ATP release [289]. Furthermore, Gx-50, a plant derivative capable of activating α7nAChR, inhibited the release of IL-1β stimulated by Aβ_1–42_ in BV2 microglia possibly through modulation of the JAK2/STAT3 and PI3K/Akt pathways [290]. Recently it was demonstrated that choline alphoscerate (also known as glyceryl phosphorylcholine, a phospholipid with high choline content) inhibited multiple effects of exposure to oligomeric Aβ_1–42_ in BV2 microglia, including reduced viability (MTT assay), increased expression of CD86 and TNF-α, and decreased expression of CD68 and IL-10 [291]. Suppression of the effects of choline alphoscerate by α-BTX pointed to an α7nAChR-dependent mechanism. Thus, signaling through the α7nAChR appears to mitigate the pro-inflammatory effects of Aβ treatment in microglia.

Stimulation of microglial α7nAChR has also been reported to modulate phagocytosis of Aβ. The selective α7nAChR agonists DMXBA (also known as GTS-21) and PNU-282987 promoted Aβ phagocytosis in rat primary microglia, and this was dependent on α7nAChR but not α4β2nAChR. In brain sections of APP/PS1dE9 mice treated with DMXBA for 56 days, the amount of Aβ within microglia was increased relative to untreated animals, and the number of microglia surrounding Aβ plaques was also elevated [292]. These changes were accompanied by improvements in water maze performance, although the performance improvements may not be attributed solely to effects of DMXBA on microglial phagocytosis. In addition, treatment with nicotine or galantamine enhanced Aβ phagocytosis in primary rat microglia, an effect which was blocked by MLA [293]. Although galantamine acts as an acetylcholinesterase (AChE) inhibitor, it also functions as a positive allosteric modulator of nicotinic receptors, serving to sensitize nAChR responding to ACh [294]. The effect of galantamine was dependent on the presence of choline in the culture medium, consistent with the action of galantamine at the allosteric binding site [293]. This suggests an additional mechanism through which galantamine may provide benefit in the AD brain [293]. However, the authors note that in the absence of galantamine, only quite high concentrations of choline were capable of stimulating amyloid phagocytosis; therefore under physiological conditions it is unlikely that choline acting through α7nAChR plays a significant role in amyloid phagocytosis.

While effects of α7nAChR on microglial inflammatory responses induced by Aβ suggest that modulation of the receptor is beneficial, the role of endogenous α7nAChR signaling in mediating damaging effects of Aβ or in protecting against these effects is uncertain. However, reduced cholinergic input to the hippocampus and cortex in AD might be expected to impair α7nAChR-dependent regulation of anti-inflammatory responses.

Unfortunately, very little is known about changes in the expression of microglial α7nAChR in the AD brain. In APP/PS1 mice, the percentage of α7-positive microglia increased in the cortex and hippocampus at 6 months compared to 3 months (coinciding with plaque deposition) but decreased thereafter [295]. However, no comparison was made to wildtype mice in this study. Velazquez et al. [296] reported an increase in the co-expression of α7nAChR and the microglial marker Iba-1 compared to non-transgenic controls at ~10 months of age. What effects this may have and whether microglia show changes in α7nAChR expression in the human AD brain is unknown.

## 16. Effects of α7nAChR Knockout on AD Pathology In Vivo

If neuronal and/or glial α7nAChR play a role in the pathology of AD, it might be expected that knockout of the receptor would affect pathological processes associated with the disease in vivo. Consistent with a physiological role for α7nAChR in mediating processes underlying learning and memory, α7-subunit-knockout mice exhibit deficits in various behavioral assays, including delayed matching to place [297], five-choice serial-reaction time task [298], radial arm maze [299], and novel object location and recognition tasks [300]. α7 knockout mice also showed an age-dependent impairment in LTP in CA3-CA1 synapses [300]. These results suggest that reduced α7nAChR expression in AD (especially in the context of reduced BFCS input) could contribute to cognitive deficits. Intriguingly, though, α7 knockout mice also show AD-related pathology, including increased Aβ_1–42_ in the hippocampus, elevated tau phosphorylation, formation of paired helical filaments and neurofibrillary tangles, and CA1 neuron loss. These results suggest that, under normal conditions, the receptor may suppress these pathological processes. Furthermore, α7 knockout exacerbated cognitive deficits in pre-plaque-depositing Tg2576 mice and led to increased soluble oligomeric Aβ, as well as enhanced neuron loss in the hippocampus [301]. In this study, behavior and neuropathology were assessed in young (5 month old) mice. The worsened pathology and cognitive function in α7 knockout mice suggest that, at least at this early stage, α7nAChR may serve a neuroprotective function against damage induced by Aβ [301].

In contrast, crossing α7 knockout mice with PDAPP mice overexpressing human mutant APP led to improved memory on the Morris water maze compared to PDAPP mice with normal α7 expression, along with preserved synaptophysin levels in the cortex and hippocampus and reversal of LTP deficits [302]. These experiments were performed in older mice and suggest that the Aβ/α7nAChR interaction may contribute to AD pathology and cognitive deficits expressed at later stages.

Whether the discrepant results of these in vivo studies relate to the use of different animal models or the age of the animals is unknown. The idea that α7nAChR interaction with Aβ may counteract the effects of Aβ at early stages but participate in processes that lead to neuronal dysfunction and cognitive decline at later stages of disease is intriguing and suggests that efforts to modulate α7nAChR as a therapeutic strategy should take disease stage into account. In both of these models, time course studies would be informative to determine whether the influence of α7 knockout varies with age in the same mouse strain. In addition, conditional knockouts in which α7 function is selectively lost at different ages would be of great value. Furthermore, α7 knockout in select cell populations would enable the separate contribution of neuronal and glial α7nAChR to Aβ-induced pathology and cognitive deficits to be assessed. Finally, use of animal models bearing both amyloid and tau pathology in these types of studies will be important to better understand the links between amyloid, α7nAChR, and tau. For example, while chronic nicotine treatment reduced plaque load in APP mice [303], it increased tau phosphorylation in 3xTg mice [83].

## 17. Conclusions

Under normal conditions, α7nAChR support physiological functions of low Aβ concentrations in neurons and glia. During early stages of disease, increased levels of Aβ disrupt the cholinergic control of circuits which normally exist under a tightly regulated balance of excitation and inhibition. This may occur through inhibition of α7nAChR on GABAergic neurons that provide inhibitory input to excitatory cells or through presynaptic modifications leading to increased glutamate release onto postsynaptic excitatory cells. Aβ-induced α7nAChR-dependent release of glutamate from nearby astrocytes could exacerbate the excessive excitatory drive and lead to increased noise in the circuit by activation of eNMDARs. Neuronal upregulation of α7nAChR in response to chronic exposure to Aβ might also contribute to hyperexcitability. Excessive intracellular Ca^2+^ associated with hyperexcitability stimulates excitotoxic signaling cascades leading to neuron loss. At later stages of disease, α7 receptors are downregulated, and the brain becomes progressively hypoactive. Whether and how hypoactivity relates to decreased α7nAChR expression is not known. Although hypoactivity may somehow be induced as a response to α7nAChR stimulation earlier in the disease course, the mechanisms linking these processes have not been identified.

Variability in the source, peptide species (e.g., 1–40 vs. 1–42), preparation methods, and consequently, the aggregation state of β-amyloid renders the interpretation of all of the above data quite complex. Aβ oligomers exist in a variety of sizes and conformations, which may operate through different mechanisms to contribute differentially to diverse neuropathological processes [93,304,305,306,307]. The conformation of Aβ used in many of the aforementioned studies is not clear, and this may contribute to discrepancies between results. Even when the size or conformation of Aβ oligomers are characterized, preparations contain a mixture of species and the species responsible for mediating particular actions is difficult to tease apart. Furthermore, the experimental systems used to investigate α7nAChR/Aβ interactions have varied widely and the degree to which these systems mimic the in vivo interaction of Aβ with α7nAChR and faithfully reproduce the downstream effects is unknown. Nevertheless, the existing literature indicates that, while low, physiological (picomolar to low nanomolar) concentrations of β-amyloid act at least in part through α7nAChR to facilitate synaptic transmission and plasticity, higher concentrations dysregulate α7nAChR function, leading to neuronal dysfunction.

α7nAChR has been proposed as a therapeutic target for AD, but despite successful preclinical development of several α7nAChR ligands [42], none have succeeded in clinical trials. Given the role of Aβ/α7nAChR interaction in mediating normal physiological functions of Aβ, the possibility that α7nAChR plays different roles at different stages of the disease, the complexity arising from expression of the receptor in multiple neuronal and non-neuronal cell populations, the dynamic regulation of receptor expression in response to ligand exposure, and even uncertainty as to whether agonism, antagonism, or allosteric modulation of α7nAChR is the appropriate strategy, it will be challenging to effectively target the right therapeutic to the right cells in the right place at the right time. In addition to receptor ligands, other methods of modulating the α7nAChR interaction with Aβ should be explored. However, development of potential therapeutics will benefit from continued exploration of the role of α7nAChR in mediating both physiological and pathological effects of Aβ.

## Figures and Tables

**Table 1:** Summary of α7nAChR changes in AD brain.

Brain Region	Method	Results	Reference
frontal cortex (superior frontal gyrus)	ISH	no change	[60]
frontal cortex (superior frontal gyrus)	ISH	no change	[57]
frontal cortex (anterior cingulate)	α-BTX binding (AR)	no change	[52]
frontal cortex	α-BTX binding (homogenates)	no change	[50]
frontal cortex	α-BTX binding (homogenates)	no change	[53]
frontal cortex (mid-frontal cortex)	α-BTX binding (homogenates)	no change	[54]
frontal cortex (superior frontal cortex)	MLA binding (homogenates)	no change (MCI or mild-moderate AD)	[71]
frontal cortex	WB	decreased in AD	[58]
frontal cortex	IHC	decreased in AD (and negatively correlated with Aβ)	[72]
frontal cortex (superior frontal gyrus)	IHC	decreased labeling intensity and reduced density of α7 protein-expressing neurons, ratio of strongly α7 protein-expressing to α7 mRNA-expressing cells was reduced in AD compared to controls	[60]
frontal cortex (superior frontal gyrus)	IHC	decreased labeling intensity and decrease in the number of α7 immunoreactive neurons in AD	[57]
hippocampus	mRNA	increased in AD	[49]
hippocampus	α-BTX binding (homogenate membranes)	decreased in AD	[49]
hippocampus	α-BTX binding (AR)	no change	[52]
hippocampus and subiculum	α-BTX binding (AR)	no change	[73]
hippocampus	WB	decreased in AD	[55]
hippocampus	IHC	increased % of astrocytes expressing α7 in familial and sporadic AD compared to age-matched controls	[51]
hippocampus	IHC	decreased labeling intensity and number of α7-positive neurons in AD	[51]
hippocampus	IHC	decreased in AD (and negatively correlated with Aβ)	[72]
hippocampus	IHC	increased % of astrocytes expressing α7 in AD	[74]
hippocampus	IHC	no change in α7-positive neurons in CA1, CA2, CA3, CA4, and entorhinal cortex and decrease in layer 2 subiculum.	[75]
hippocampus	IHC	increased number of α7-positive astrocytes throughout hippocampal layers, subiculum, and entorhinal cortex in AD	[75]
temporal cortex	mRNA	no change	[76]
temporal cortex (mid-temporal gyrus)	α-BTX binding (homogenates)	decreased in AD	[50]
temporal cortex	α-BTX binding (AR)	no change	[52]
temporal cortex	α-BTX binding (homogenate membranes)	no change	[49]
temporal cortex	α-BTX binding (homogenate membranes)	decreased in familial AD (APPswe) but not sporadic AD compared to respective age-matched controls	[51]
temporal cortex	WB	no change	[59]
temporal cortex (superior temporal gyrus)	WB	decreased in AD	[56]
temporal cortex (superior temporal gyrus)	WB	decreased in AD	[57]
temporal cortex	WB	no change	[55]
temporal cortex	IHC	number of α7+ cells reduced in AD (but remaining positive neurons exhibit intense labeling)	[61]
temporal cortex (entorhinal cortex)	IHC	increased % of astrocytes expressing α7 in AD	[74]
temporal cortex (entorhinal cortex)	IHC	increased α7 content in astrocytes in AD	[77]
temporal cortex	IHC	increased % of astrocytes expressing α7nAChR in familial and sporadic AD compared to age-matched controls (and higher in familial than sporadic AD).	[51]
temporal cortex	IHC	decreased labeling intensity and number of α7-positive neurons in AD	[51]
temporal cortex	IHC	decreased in AD (and negatively correlated with Aβ)	[72]
nucleus basalis	mRNA (single cell and tissue homogenates)	no change in MCI, increased in mild-moderate AD compared to controls and MCI and inversely correlated with cognitive test scores	[62]
caudate nucleus	mRNA (homogenates)	no change in MCI or mild-moderate AD	[62]
cerebellum	mRNA	no change	[49]
cerebellum	α-BTX binding (membranes)	increased in AD	[49]
cerebellum	WB	no change	[58]
multiple	18F-ASEM PET	increased availability in multiple brain regions in MCI (striatum, hippocampus, temporal cortex, occipital cortex, cingulate cortex, frontal cortex, parietal cortex, cerebellum, and basal forebrain)	[70]

AR: autoradiography; IHC: immunohistochemistry; ISH: in situ hybridization; WB: Western blot.

## Abbreviations

The following abbreviations are used in this manuscript:

ACh	acetylcholine
AChE	acetylcholinesterase
AD	Alzheimer’s disease
ApoE	apolipoproteinE
APP	amyloid precursor protein
AR	autoradiography
ATP	adenosine triphosphate
Aβ	beta amyloid
α-BTX	alpha-bungarotoxin
BACE1	beta-site amyloid precursor protein cleaving enzyme 1
BFCS	basal forebrain cholinergic system
CA1	cornu ammonis 1
CA3	cornu ammonis 3
CaMKII	Ca^2+^/calmodulin-dependent protein kinase II
CAP	cholinergic anti-inflammatory pathway
CD	cluster of differentiation
E-LTP	early long-term potentiation
eNMDAR	extrasynaptic NMDA receptor
EphB2	ephrin type-B receptor 2
ERK	extracellular signal-regulated kinase
GABA	gamma-aminobutyric acid
GCLC	glutamate-cysteine ligase catalytic subunit
GLAST	glutamate aspartate transporter
GSK	glycogen synthase kinase
HO1	heme oxygenase I
IHC	immunohistochemistry
IkBa	NF-κB inhibitor alpha
IL	interleukin
IP3	inositol triphosphate
ISH	in situ hybridization
JAK2	Janus kinase 2
L-LTP	late-long-term potentiation
LPS	lipopolysaccharide
LTD	long-term depression
LTP	long-term potentiation
mAChR	muscarinic acetylcholine receptor
MAPK	mitogen-activated protein kinase
MCI	mild cognitive impairment
mEPSC	miniature excitatory postsynaptic current
MIP-1α	macrophage inflammatory protein-1 alpha
MLA	methyllycaconitine
MPP+	1-methyl-4-phenylpyridinium
nAChR	nicotinic acetylcholine receptor
NADPH	nicotinamide adenine dinucleotide phosphate
NF-kB	nuclear factor-kappa B
NGF	nerve growth factor
NgR1	nogo receptor 1
NMDA	N-methyl-D-aspartate
Nrf-2	nuclear factor erythroid 2–related factor 2
P2X7R	purinergic P2X7 receptor
p75NTR	p75 neurotrophin receptor
PAMP	pathogen-associated molecular pattern
PET	positron emission tomography
PI3K	phosphoinositide 3-kinase
PKA	protein kinase A
PKC	protein kinase C
PLC	phospholipase C
PP2B	Serine/threonine protein phosphatase 2B (calcineurin)
Prpc	cellular prion protein
PS1	presenilin 1
RAGE	receptor for advanced glycation end products
RANTES	regulated on activation, normal T cell expressed and secreted
ROS	reactive oxygen species
shRNA	short hairpin RNA
STAT3	signal transducer and activator of transcription 3
STEP	Striatal-enriched phosphatase
TNF	tumor necrosis factor
TR-FRET	time-resolved fluorescence energy transfer
TXNRD1	thioredoxin reductase 1
VGCC	voltage-gated Ca^2+^ channel
WB	Western blot
